# A phosphorylation map of the photosystem II supercomplex C2S2M2

**DOI:** 10.3389/fpls.2013.00459

**Published:** 2013-11-15

**Authors:** Sujith Puthiyaveetil, Helmut Kirchhoff

**Affiliations:** Institute of Biological Chemistry, Washington State UniversityPullman, WA, USA

**Keywords:** PS II core phosphorylation, PS II repair cycle, photoinhibition, PS II supercomplex, state transitions, LHC II phosphorylation, Stn8, Stn7

## PS II phosphorylation

It was 36 years ago when John Bennett first discovered chloroplast protein phosphorylation (Bennett, 1977). The most conspicuous chloroplast phosphoprotein was the apoprotein of the Light Harvesting Complex II (LHC II) of photosystem II (PS II). Other phosphoproteins included the reaction center protein CP43 and the quinone-binding proteins D1 and D2 of PS II. These earlier studies also identified the PsbH protein, the minor subunit of PS II, as another chloroplast phosphoprotein (Allen, 1992). Nearly all of the first-known chloroplast phosphoproteins belong to PS II, the water-oxidizing photosystem of oxygenic photosynthesis.

With more than 25 protein subunits and 350 kDa in size, the PS II core monomer is a massive structure (Umena et al., 2011). This enormous size is in addition to the peripheral light harvesting complexes. The major peripheral antenna of PS II is LHC II, which is a trimer. Some, so-called minor, LHC are monomeric and act as linkers in connecting the trimeric LHC II to the core. The core is composed of the reaction center proteins D1 and D2 and an internal antenna made up of CP43 and CP47. The PS II core with its peripheral antenna forms supercomplexes, which mainly differ in the number of LHC II bound to the core (Caffarri et al., 2009). The core itself is dimeric (C2) to which the LHC II could strongly (S), or moderately (M), or loosely (L) bind. The monomeric linker associated with the S-form is CP26, and for the M-form are CP29 and CP24. A dimeric core (C2) with 2 S and 2 M LHC II (C2S2M2) is the most abundant supercomplex in plant photosynthetic membranes (Kouril et al., 2012).

PS II phosphorylation is light dependent. It turned out, however, light acts through the redox state of the plastoquinone (PQ) pool (Allen et al., 1981; Bennett, 1991). PS II phosphorylation can be divided into the core phosphorylation (D1, D2, CP43, and PsbH) and the peripheral antenna phosphorylation (LHC II). In plants, the protein kinases that phosphorylate the core and the LHC II have been identified as Stn8 and Stn7, respectively, (Bellafiore et al., 2005; Bonardi et al., 2005). PS II phosphorylation is only observed in green algae and plants, and not in cyanobacteria and red algae where the light harvesting antenna is phycobilisome-based (Pursiheimo et al., 1998). Core phosphorylation is responsive to light intensity with increasing levels of phosphorylation observed in high light (Elich et al., 1992; Tikkanen and Aro, 2012). On the contrary, LHC II phosphorylation was restricted to low light condition specific to PS II (Rintamaki et al., 2000). At higher light intensities, the activity of the Stn7 is inhibited by the reduced stromal electron carrier thioredoxin (Rintamaki et al., 2000; Puthiyaveetil, 2011). It was also noted that only the L-form of the LHC II trimer is phosphorylated, while the LHC II isoforms comprising the S- and M-trimers are not phosphorylated or do not contain phosphorylation sites (Galka et al., 2012). LHC II phosphorylation is the basis of state transitions, an acclimatory response to changes in light quality (Bonaventura and Myers, 1969; Murata, 1969). The precise function of PS II core phosphorylation, however, is still unclear. PS II undergoes damage in light, which results in photoinhibition—light-induced loss of photosynthetic activity (Aro et al., 1993; Long et al., 1994).

In plants and green algae, PS II is mainly found in the stacked granal regions of the thylakoid membrane where it forms almost immobile supercomplexes (Kirchhoff et al., 2008; Mullineaux, 2008). The centrally located reaction center protein D1 is the main target of photodamage in PS II (Kyle et al., 1984). To maintain photosynthetic efficiency in high light, chloroplasts have evolved a robust repair process known as the PS II repair cycle, wherein the damaged D1 protein is degraded and replaced with a newly synthesized copy (Melis, 1999; Nixon et al., 2010). The PS II repair cycle operates through many constraints (Kirchhoff, 2013b), chief among them are how the repair machinery located in the unstacked regions of the thylakoid membrane accesses the damaged photosystems in the stacked, crowded granal membranes and how the damaged photosystems are mobilized in the rather “immobile” grana. The proposed functions of PS II phosphorylation include “marking” the damaged photosystems for degradation (Aro et al., 1993), increasing the mobility of photosystems (Goral et al., 2010; Herbstova et al., 2012), disassembly of PS II (Tikkanen et al., 2008; Fristedt and Vener, 2011), decreasing the granal diameter (Herbstova et al., 2012; Kirchhoff, 2013a), and for maintaining electron flow in high light (Harrison and Allen, 1991). Some of these proposals are not mutually exclusive. Functions such as increased mobility and disassembly are especially supported but how precisely phosphorylation produces these effects are unclear.

## Mapping phosphorylation sites onto the PS II supercomplex C2S2M2

Significant advances in mass-spectrometry-based phosphoproteomics approaches have enabled the mapping of phosphorylation sites in PS II proteins (Turkina et al., 2006; Lemeille and Rochaix, 2010; Reiland et al., 2011). To gain insight into the function of PS II core phosphorylation, we projected known phosphorylation sites on the PS II supercomplex C2S2M2. The available PS II atomic coordinates, however, are for cyanobacteria (Umena et al., 2011) and not for plants where PS II phosphorylation actually occurs. Furthermore, the mapped phosphorylation sites are for *Arabidopsis* and *Chlamydomonas* proteins. For the monomeric antenna CP29, however, structure from a plant source is available (Pan et al., 2011). LHC II also has a structure solved from spinach (Liu et al., 2004). In our approach, we therefore, aligned the amino acid sequence of the *Arabidopsis* PS II core phosphoproteins with that of cyanobacteria and then projected the positions of phosphorylation sites on the cyanobacterial sequence and structure. For PS II core proteins, phosphorylation sites are located usually on their stromaly-positioned N-termini. Even though homology exists between N-termini of cyanobacterial and *Arabidopsis* proteins and the information on phosphorylation site positions can safely be transferred between them, the structure of their N-termini has not been resolved to their entire length for some cyanobacterial proteins (Umena et al., 2011). Under these circumstances, the nearest amino acid on the N-terminus, for which structural information exists, is taken as the approximate position of the phosphorylation site.

For the *Arabidopsis* D1 and D2 proteins, threonine 2 is the phosphorylation site and for *Arabidopsis* CP43, threonine 15 (Reiland et al., 2011). All three sites are conserved in *Chlamydomonas* and cyanobacterial proteins. For PsbH, multiple phosphorylation sites have been noted as threonines 3 and 5 (Reiland et al., 2011), both of which are conserved in *Chlamydomonas* but not in cyanobacteria. The monomeric antenna CP29 has at least five high light-dependent phosphorylation sites, only one of which, threonine 37 in Lhcb4.2 isoform, is catalyzed by the Stn8 kinase (Fristedt and Vener, 2011), while others are catalyzed by Stn7.

**Figure 1:**
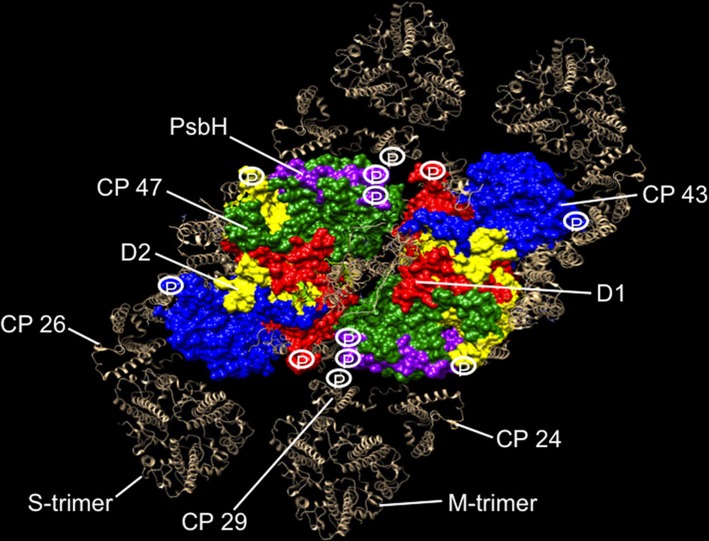
**Phosphorylation map of the C2S2M2 supercomplex as catalyzed by the Stn8 kinase**. C2S2M2 rendered by the Chimera visualization program where the view is from the stromal side looking down. Coordinates for PS II core were taken from the pdb structure 3ARC (Umena et al., 2011), LHC II from 1RWT (Liu et al., 2004), and CP29 from 3PL9 (Pan et al., 2011). Since no structures were available for CP26 and CP24, CP29 coordinates were used instead. Supercomplex structure was modeled as in Caffarri et al. (2009). Major subunits of the core and the complete peripheral antenna are shown. Individual subunits of the core have been differently colored and labeled. The peripheral antenna is shown in silver and labeled. Approximate positions of phosphorylation sites of D1, D2, CP43, PsbH, and CP29 are shown. For CP29, up to five high light-dependent phosphorylation sites are known (Fristedt and Vener, 2011), but only the Stn8 phosphosite is shown for consistency. Phosphosites have also been reported for the other two monomeric antennas CP24 and CP26, but the identity of their kinases and the light condition in which they become phosphorylated are uncertain (Reiland et al., 2011), hence not included in our analysis.

Atomic coordinates for *Thermosynechococcus vulcanus* PS II core proteins D1, D2, CP43, CP47, and PsbH are rendered in a space-filling model using the Chimera program (Pettersen et al., 2004) (Figure 1). The monomeric and trimeric antenna coordinates are rendered by the ribbon diagram, and modeled around the core from positions determined by the single particle analysis (Caffarri et al., 2009). The phosphorylation sites of PS II proteins are then projected onto the supercomplex structure to reveal the distribution of phosphosites in relation to PS II proteins (Figure 1).

## The function of PS II core phosphorylation

Our projections reveal strategic location of phosphosites around the periphery of the core PS II, where the monomeric antennas CP29, CP24, and CP26 attach and at the monomer-monomer interface (Figure 1). From this distribution, we suggest that the function of PS II core phosphorylation is antenna dissociation and monomerization of the dimeric core. Phosphorylation-mediated antenna dissociation is likely to be the mechanism by which the S- and M-trimers are removed from the core. It remains to be determined how, in molecular terms, phosphorylation causes antenna dissociation and core monomerization.

## PS II phosphorylation in PS II repair cycle

The primary purpose of antenna dissociation will be to relieve the excitation pressure on PS II reaction centers and to prevent further damage to D1 in high light. The detached antenna, however, unless in a quenched state, can be a source of destructive reactive oxygen species. An antenna supercomplex dubbed as “band 4,” comprising LHC II, CP29, and CP24 has been suggested as the site of protective non-photochemical quenching (NPQ) in high light (Betterle et al., 2009). Interestingly, this supercomplex is precisely what our model of phosphorylation-mediated antenna dissociation would generate during disassembly (Figure 1). However, the apparent correlation between phosphorylation and the band 4-mediated NPQ is yet to be investigated.

As discussed earlier, our model predicts that PS II core phosphorylation drives monomerization of the dimeric cores. Monomerization has also been suggested to result from the damage or rearrangement of the extrinsic PsbO subunit of PS II (Nixon et al., 2010). If this is indeed the case, phosphorylation might act alone or in conjunction with PsbO in causing monomerization. As a consequence of antenna dissociation and core monomerization, larger supercomplexes will be broken up into smaller PS II particles. The observation that most of the PS II supercomplexes and PS II core dimers remain intact in high light in the absence of phosphorylation is consistent with our proposal (Tikkanen et al., 2008; Nath et al., 2013).

During the PS II repair cycle, the following scenario can be envisioned. PS II is phosphorylated, whether they are damaged or not. This will break up supercomplexes into monomeric cores. Monomeric cores in the grana, with their smaller particle size, will be able to diffuse faster toward the stromaly-exposed regions of the thylakoid membrane where the repair machinery has access to the damaged photosystems. A swift migration of the monomeric cores may also result from the smaller diffusion area of the grana as they shrink in diameter in high light (Herbstova et al., 2012; Kirchhoff, 2013a). Following dephosphorylation and the partial disassembly of the PS II monomer into the so-called “RC47 complex,” proteases recognize damaged PS II by conformational changes brought about by the damage and degrade them. The newly synthesized and processed D1 is then inserted, activated, and assembled by the repair machinery. The resultant PS II core monomer will diffuse back to the grana where they could dimerize and be fitted with their new antenna. Similarly, the phosphorylated but undamaged PS II core monomer, following dephosphorylation, could simply slip back to the grana, dimerize, and reassemble with their antenna.

## Antenna dissociation in state transitions and PS II repair cycle: a common regulatory theme

State transitions correct imbalance in excitation of individual photosystems by redistributing excitation energy to the rate-limiting photosystem (Bonaventura and Myers, 1969; Murata, 1969; Allen, 2003). When PS II is preferentially illuminated, LHC II gets phosphorylated and the phospho-LHC II dissociates from PS II. It then associates with photosystem I (PS I) as its light harvesting antenna. When dephosphorylated, LHC II re-joins PS II from PS I. Phosphorylation of LHC II is thought to change its molecular recognition of PS II (Allen and Forsberg, 2001). The similarity of phosphorylation-mediated antenna dissociation in state transitions and PS II repair is thus unmistakable and supports antenna dissociation as a unifying function of PS II phosphorylation.

This functional similarity of LHC II and PS II core phosphorylation is even deeper. The protein kinases, Stn7 and Stn8, which underpin state transitions and PS II repair, are paralogs, sharing 43% identity at the amino acid sequence level. They both require reduced PQ for activation (Allen et al., 1981; Bennett, 1991). Studies of the kinase mutants reveal significant substrate overlap between the kinases (Depege et al., 2003; Bellafiore et al., 2005; Bonardi et al., 2005; Puthiyaveetil et al., 2012), suggesting their ancient origin from gene duplication and subsequent divergence. Even though the Stn7 kinase is inhibited in high light, its role in PS II disassembly through CP29 phosphorylation in the early stages of high light acclimation cannot be ruled out (Fristedt and Vener, 2011). Phosphorylation-mediated detachment of CP29 from PS II is also a feature of green algal state transitions (Kargul et al., 2005; Takahashi et al., 2006). Significant functional and amino acid sequence similarity exists also for the LHC II and PS II core phosphatases (Pribil et al., 2010; Shapiguzov et al., 2010; Samol et al., 2012). Minimizing PS II excitation pressure through antenna dissociation thus appears to be the defining feature of phosphorylation in state transitions and in PS II repair cycle.

## Author contributions

Sujith Puthiyaveetil and Helmut Kirchhoff wrote the paper.

## References

[B1] Allen J. F. (1992). Protein phosphorylation in regulation of photosynthesis. Biochim. Biophys. Acta 1098, 275–335 10.1016/S0005-2728(09)91014-3 1310622

[B2] Allen J. F. (2003). State transitions—a question of balance. Science 299, 1530–1532 10.1126/science.1082833 12624254

[B3] Allen J. F. Bennett J. Steinback K. E. Arntzen C. J. (1981). Chloroplast protein phosphorylation couples plastoquinone redox state to distribution of excitation-energy between photosystems. Nature 291, 25–29 10.1038/291025a016245115

[B4] Allen J. F. Forsberg J. (2001). Molecular recognition in thylakoid structure and function. Trends Plant Sci. 6, 317–326 10.1016/S1360-1385(01)02010-6 11435171

[B5] Aro E. M. Virgin I. Andersson B. (1993). Photoinhibition of photosystem II. Inactivation, protein damage and turnover. Biochim. Biophys. Acta 1143, 113–134 10.1016/0005-2728(93)90134-2 8318516

[B6] Bellafiore S. Barneche F. Peltier G. Rochaix J. D. (2005). State transitions and light adaptation require chloroplast thylakoid protein kinase STN7. Nature 433, 892–895 10.1038/nature03286 15729347

[B7] Bennett J. (1977). Phosphorylation of chloroplast membrane polypeptides. Nature 269, 344–346 10.1038/269344a0

[B8] Bennett J. (1991). Protein-phosphorylation in green plant chloroplasts. Annu. Rev. Plant Physiol. Plant Mol. Biol. 42, 281–311 10.1146/annurev.pp.42.060191.001433

[B9] Betterle N. Ballottari M. Zorzan S. De Bianchi S. Cazzaniga S. Dall'osto L. (2009). Light-induced dissociation of an antenna hetero-oligomer is needed for non-photochemical quenching induction. J. Biol. Chem. 284, 15255–15266 10.1074/jbc.M808625200 19307183 PMC2685706

[B10] Bonardi V. Pesaresi P. Becker T. Schleiff E. Wagner R. Pfannschmidt T. (2005). Photosystem II core phosphorylation and photosynthetic acclimation require two different protein kinases. Nature 437, 1179–1182 10.1038/nature04016 16237446

[B11] Bonaventura C. Myers J. (1969). Fluorescence and oxygen evolution from Chlorella pyrenoidosa. Biochim. Biophys. Acta 189, 366–383 10.1016/0005-2728(69)90168-6 5370012

[B12] Caffarri S. Kouril R. Kereiche S. Boekema E. J. Croce R. (2009). Functional architecture of higher plant photosystem II supercomplexes. EMBO J. 28, 3052–3063 10.1038/emboj.2009.232 19696744 PMC2760109

[B13] Depege N. Bellafiore S. Rochaix J. D. (2003). Rote of chloroplast protein kinase Stt7 in LHCII phosphorylation and state transition in Chlamydomonas. Science 299, 1572–1575 10.1126/science.1081397 12624266

[B14] Elich T. D. Edelman M. Mattoo A. K. (1992). Identification, characterization, and resolution of the *in vivo* phosphorylated form of the D1 photosystem II reaction center protein. J. Biol. Chem. 267, 3523–3529 1737803

[B15] Fristedt R. Vener A. V. (2011). High light induced disassembly of photosystem II supercomplexes in Arabidopsis requires STN7-dependent phosphorylation of CP29. PLoS ONE 6:e24565 10.1371/journal.pone.0024565 21915352 PMC3168523

[B16] Galka P. Santabarbara S. Khuong T. T. Degand H. Morsomme P. Jennings R. C. (2012). Functional analyses of the plant photosystem I-light-harvesting complex II supercomplex reveal that light-harvesting complex II loosely bound to photosystem II is a very efficient antenna for photosystem I in state II. Plant Cell 24, 2963–2978 10.1105/tpc.112.100339 22822202 PMC3426126

[B17] Goral T. K. Johnson M. P. Brain A. P. Kirchhoff H. Ruban A. V. Mullineaux C. W. (2010). Visualizing the mobility and distribution of chlorophyll proteins in higher plant thylakoid membranes: effects of photoinhibition and protein phosphorylation. Plant J. Cell Mol. Biol. 62, 948–959 10.1111/j.1365-313X.2010.04207.x 20230505

[B18] Harrison M. A. Allen J. F. (1991). Light-dependent phosphorylation of photosystem-ii polypeptides maintains electron-transport at high light-intensity—separation from effects of phosphorylation of LHC-Ii. Biochim. Biophys. Acta 1058, 289–296 10.1016/S0005-2728(05)80249-X

[B19] Herbstova M. Tietz S. Kinzel C. Turkina M. V. Kirchhoff H. (2012). Architectural switch in plant photosynthetic membranes induced by light stress. Proc. Natl. Acad. Sci. U.S.A. 109, 20130–20135 10.1073/pnas.1214265109 23169624 PMC3523818

[B20] Kargul J. Turkina M. V. Nield J. Benson S. Vener A. V. Barber J. (2005). Light-harvesting complex II protein CP29 binds to photosystem I of Chlamydomonas reinhardtii under State 2 conditions. FEBS J. 272, 4797–4806 10.1111/j.1742-4658.2005.04894.x 16156798

[B21] Kirchhoff H. (2013a). Architectural switches in plant thylakoid membranes. Photosyn. Res. 116, 481–487 10.1007/s11120-013-9843-0 23677426

[B22] Kirchhoff H. (2013b). Structural constraints for protein repair in plant photosynthetic membranes. Plant Signal. Behav. 18:e23634 10.4161/psb.23634 23333974 PMC7030307

[B23] Kirchhoff H. Haferkamp S. Allen J. F. Epstein D. B. Mullineaux C. W. (2008). Protein diffusion and macromolecular crowding in thylakoid membranes. Plant Physiol. 146, 1571–1578 10.1104/pp.107.115170 18287489 PMC2287334

[B24] Kouril R. Dekker J. P. Boekema E. J. (2012). Supramolecular organization of photosystem II in green plants. Biochim. Biophys. Acta 1817, 2–12 10.1016/j.bbabio.2011.05.024 21723248

[B25] Kyle D. J. Ohad I. Arntzen C. J. (1984). Membrane protein damage and repair: selective loss of a quinone-protein function in chloroplast membranes. Proc. Natl. Acad. Sci. U.S.A. 81, 4070–4074 10.1073/pnas.81.13.4070 16593483 PMC345370

[B26] Lemeille S. Rochaix J. D. (2010). State transitions at the crossroad of thylakoid signalling pathways. Photosyn. Res. 106, 33–46 10.1007/s11120-010-9538-8 20217232

[B27] Liu Z. Yan H. Wang K. Kuang T. Zhang J. Gui L. (2004). Crystal structure of spinach major light-harvesting complex at 2.72 A resolution. Nature 428, 287–292 10.1038/nature02373 15029188

[B28] Long S. P. Humphries S. Falkowski P. G. (1994). Photoinhibition of Photosynthesis in Nature. Annu. Rev. Plant Physiol. Plant Mol. Biol. 45, 633–662 10.1146/annurev.pp.45.060194.003221

[B29] Melis A. (1999). Photosystem-II damage and repair cycle in chloroplasts: what modulates the rate of photodamage? Trends Plant Sci. 4, 130–135 10.1016/S1360-1385(99)01387-4 10322546

[B30] Mullineaux C. W. (2008). Factors controlling the mobility of photosynthetic proteins. Photochem. Photobiol. 84, 1310–1316 10.1111/j.1751-1097.2008.00420.x 18764904

[B31] Murata N. (1969). Control of excitation transfer in photosynthesis. I. Light-induced changes of chlorophyll a fluorescence in Porphyridium cruentum. Biochim. Biophys. Acta 172, 242–251 10.1016/0005-2728(69)90067-X 5775694

[B32] Nath K. Poudyal R. S. Eom J. S. Park Y. S. Zulfugarov I. S. Mishra S. R. (2013). Loss-of-function of OsSTN8 suppresses the photosystem (PS) II core protein phosphorylation and interferes with PSII repair mechanism in rice (Oryza sativa). Plant J. 76, 675–686 10.1111/tpj.12331 24103067

[B33] Nixon P. J. Michoux F. Yu J. Boehm M. Komenda J. (2010). Recent advances in understanding the assembly and repair of photosystem II. Ann. Bot. 106, 1–16 10.1093/aob/mcq059 20338950 PMC2889791

[B34] Pan X. W. Li M. Wan T. Wang L. F. Jia C. J. Hou Z. Q. (2011). Structural insights into energy regulation of light-harvesting complex CP29 from spinach. Nat. Struct. Mol. Biol. 18, 309–315 10.1038/nsmb.2008 21297637

[B35] Pettersen E. F. Goddard T. D. Huang C. C. Couch G. S. Greenblatt D. M. Meng E. C. (2004). UCSF Chimera–a visualization system for exploratory research and analysis. J. Comput. Chem. 25, 1605–1612 10.1002/jcc.20084 15264254

[B36] Pribil M. Pesaresi P. Hertle A. Barbato R. Leister D. (2010). Role of plastid protein phosphatase TAP38 in LHCII dephosphorylation and thylakoid electron flow. PLoS Biol. 8:e1000288 10.1371/journal.pbio.1000288 20126264 PMC2811158

[B37] Pursiheimo S. Rintamaki E. Baena-Gonzalez E. Aro E. M. (1998). Thylakoid protein phosphorylation in evolutionally divergent species with oxygenic photosynthesis. FEBS Lett. 423, 178–182 10.1016/S0014-5793(98)00088-X 9512353 PMC7164083

[B38] Puthiyaveetil S. (2011). A mechanism for regulation of chloroplast LHC II kinase by plastoquinol and thioredoxin. FEBS Lett. 585, 1717–1721 10.1016/j.febslet.2011.04.076 21557941

[B39] Puthiyaveetil S. Ibrahim I. M. Allen J. F. (2012). Oxidation-reduction signalling components in regulatory pathways of state transitions and photosystem stoichiometry adjustment in chloroplasts. Plant Cell Environ. 35, 347–359 10.1111/j.1365-3040.2011.02349.x 21554328

[B40] Reiland S. Finazzi G. Endler A. Willig A. Baerenfaller K. Grossmann J. (2011). Comparative phosphoproteome profiling reveals a function of the STN8 kinase in fine-tuning of cyclic electron flow (CEF). Proc. Natl. Acad. Sci. U.S.A. 108, 12955–12960 10.1073/pnas.1104734108 21768351 PMC3150903

[B41] Rintamaki E. Martinsuo P. Pursiheimo S. Aro E. M. (2000). Cooperative regulation of light-harvesting complex II phosphorylation via the plastoquinol and ferredoxin-thioredoxin system in chloroplasts. Proc. Natl. Acad. Sci. U.S.A. 97, 11644–11649 10.1073/pnas.180054297 11005828 PMC17254

[B42] Samol I. Shapiguzov A. Ingelsson B. Fucile G. Crevecoeur M. Vener A. V. (2012). Identification of a photosystem II phosphatase involved in light acclimation in Arabidopsis. Plant Cell 24, 2596–2609 10.1105/tpc.112.095703 22706287 PMC3406908

[B43] Shapiguzov A. Ingelsson B. Samol I. Andres C. Kessler F. Rochaix J. D. (2010). The PPH1 phosphatase is specifically involved in LHCII dephosphorylation and state transitions in Arabidopsis. Proc. Natl. Acad. Sci. U.S.A. 107, 4782–4787 10.1073/pnas.0913810107 20176943 PMC2842063

[B44] Takahashi H. Iwai M. Takahashi Y. Minagawa J. (2006). Identification of the mobile light-harvesting complex II polypeptides for state transitions in Chlamydomonas reinhardtii. Proc. Natl. Acad. Sci. U.S.A. 103, 477–482 10.1073/pnas.0509952103 16407170 PMC1326185

[B45] Tikkanen M. Aro E. M. (2012). Thylakoid protein phosphorylation in dynamic regulation of photosystem II in higher plants. Biochim. Biophys. Acta 1817, 232–238 10.1016/j.bbabio.2011.05.005 21605541

[B46] Tikkanen M. Nurmi M. Kangasjarvi S. Aro E. M. (2008). Core protein phosphorylation facilitates the repair of photodamaged photosystem II at high light. Biochim. Biophys. Acta 1777, 1432–1437 10.1016/j.bbabio.2008.08.004 18774768

[B47] Turkina M. V. Kargul J. Blanco-Rivero A. Villarejo A. Barber J. Vener A. V. (2006). Environmentally modulated phosphoproteome of photosynthetic membranes in the green alga Chlamydomonas reinhardtii. Mol. Cell. Proteomics 5, 1412–1425 10.1074/mcp.M600066-MCP200 16670252

[B48] Umena Y. Kawakami K. Shen J.-R. Kamiya N. (2011). Crystal structure of oxygen-evolving photosystem II at a resolution of 1.9 angstrom. Nature 473, 55–65 10.1038/nature09913 21499260

